# The History of Prostitution

**Published:** 1899-03

**Authors:** 


### Book Reviews.

THE HISTORY OF PROSTITUTION. Its extent, causes and effects throughout the world. By WILLIAM W. SANGER, M. D., Resident Physician, Blackwell's Island, New York City; member of the American Association for the Advancement of Science; late one of the Physicians to the Marine Hospital, Quarantine, New York, etc., with numerous Editorial Notes and an Appendix. 709 pages, 8vo. New York.: The Medical Publishing Co. 1898.

No author can lay claim to a higher inspiration than that his work was conceived in the noble purpose of benefiting his fellow man; and the reader of the following pages cannot doubt that it was in this spirit and solely with this purpose that Dr. Sanger compiled and gave to the American public "The History of Prostitution."

The author has gone into this subject treating it scientifically and historically and has clothed a delicate subject with such adroitness that much of the sensationalism connected with it is overcome. The existence of prostitution among the Jews—early in the VIIIth Century, B. C.—shows that it is coeval with society. He then sketches the customs of the Egyptians, Syrians, Grecians, Romans, down to the middle ages, then the statistics of the European countries and finally a careful and painstaking consideration of the question in New York City. The book is carefully written and is well worth the attention and consideration of those who seek to remedy one of the greatest of evils confronting the human family.

The medical and legal professions, as well as the student of sociology, will find it an invaluable compilation of historical and statistical information, replete with all data embraced within the meaning of its title and indexed so as to enable the reader to find at a glance any subject desired. The book is printed on good paper and well bound. W. C. K.

